# Methanolic Extract of *Rhizophora mangle* (Rhizophoraceae) Leaves: Phytochemical Characterization and Anthelmintic Evaluation against *Schistosoma mansoni*

**DOI:** 10.3390/ph17091178

**Published:** 2024-09-06

**Authors:** Wilza W. M. França, Sérgio D. Magalhães Filho, Lucas A. O. Cavalcante, Mary A. A. S. Gomes, Maria T. V. Gonçalves, Emily G. M. Diniz, Wheverton R. C. Nascimento, Reginaldo G. Lima Neto, Mônica C. P. A. Albuquerque, Iranildo J. Cruz Filho, Hallysson D. A. Araújo, André L. Aires, Jeymesson R. C. Vieira

**Affiliations:** 1 Centro de Biociência, Programa de Pós-Graduação em Morfotecnologia, Universidade Federal de Pernambuco, Avenida Prof. Moraes Rego, 1235, Cidade Universitária, Recife 50670-501, PE, Brazil; wilza.franca@ufpe.br (W.W.M.F.); jeymesson.vieira@ufpe.br (J.R.C.V.); 2 Centro de Ciências Médicas, Programa de Pós-Graduação em Medicina Tropical, Universidade Federal de Pernambuco, Avenida Prof. Moraes Rego, 1235, Cidade Universitária, Recife 50670-501, PE, Brazil; 3 Instituto Keizo Asami (iLIKA), Universidade Federal de Pernambuco, Avenida Prof. Moraes Rego, 1235, Cidade Universitária, Recife 50670-501, PE, Brazil; 4 Centro de Ciências Médicas, Área Acadêmica de Medicina Tropical, Universidade Federal de Pernambuco, Avenida Prof. Moraes Rego, 1235, Cidade Universitária, Recife 50670-501, PE, Brazil; 5 Laboratório de Biotecnologia e Fármacos, Laboratório de Tecnologia de Biomateriais, Centro Acadêmico de Vitória de Santo Antão, Universidade Federal de Pernambuco, Vitória de Santo Antão 55608-680, PE, Brazil

**Keywords:** phytotherapy, antischistosomal agent, schistosomiasis mansoni, toxic potential praziquantel

## Abstract

*Rhizophora mangle* is commonly used in traditional medicine to treat infections, reduce inflammation, and promote healing. This study aimed to analyze the phytochemical profile of the methanolic extract of *R. mangle* leaves (MELRm) and evaluate its in vitro schistosomicidal activity against *Schistosoma mansoni* as well as its cytotoxicity. Plant material was collected in Itamaracá City, Pernambuco, Brazil. The extract was analyzed using UV/Vis spectrophotometry and high-performance liquid chromatography (HPLC). The motility, mortality, and cell viability of adult worms were assessed in a schistosomicidal assay, while cytotoxicity was evaluated through a colorimetric assay with MTT on RAW 264.7 cells. The primary compounds identified in MELRm were phenolic compounds. In the schistosomicidal assay, all concentrations of MELRs induced changes in the motility of adult worms. At a concentration of 400 μg/mL, MELRs resulted in 56.25% mortality after 72 h of incubation. After 120 h, mortality rates of 75%, 62.5%, and 50% were observed at MELRm concentrations of 400, 200, and 100 μg/mL, respectively. No eggs were detected at any MELRm concentration. MELRs did not show cytotoxicity towards RAW 264.7 cells at the concentrations tested. These results indicate that MELRs demonstrate schistosomicidal activity in vitro, suggesting they are promising candidates for in vivo studies.

## 1. Introduction

Schistosomiasis is a neglected parasitic disease caused by parasites of the genus *Schistosoma* spp., which has significant social and economic impacts [1]. The infection is prevalent among populations with low visibility and little political voice in areas with poor or non-existent basic sanitation, as well as limited access to health services and education [1,2,3,4]. Epidemiologically, around 779 million people live in areas at risk of infection, and 251.4 million are infected each year. Of those infected, 20 million develop the chronic phase of the disease, and 280,000 die annually [1,2].

For the past 50 years, praziquantel (PZQ) has been the only drug recommended by the World Health Organization (WHO) for the treatment of all types of human schistosomiasis. This measure has effectively reduced the prevalence and incidence of schistosomiasis worldwide, as there is currently no effective vaccine [3,4]. PZQ is administered orally, has low toxicity and acts only against adult worms. It is effective in treating various clinical forms of the infection [1,3,4]. These advantages, in part, contribute to the limited research efforts in the search for new schistosomal drug candidates. In endemic areas, there is commonly a shortage or even an absence of effective public health and sanitation policies for the prevention and treatment of schistosomiasis [4]. Furthermore, the number of available doses of PZQ is insufficient to meet the demand of those infected. According to the WHO, only 20% of those infected receive treatment [1].

The extensive use of PZQ in preventive and mass chemotherapy in endemic areas, the reliance on a single drug to treat this expanding disease, and reports of resistance and/or tolerance in strains of *Schistosoma* spp. to PZQ are concerns for the medical and scientific communities. These issues underscore the need for research into new pharmacological alternatives for schistosomiasis, including those derived from plants [3,5,6]. Mangrove ecosystems are attractive for prospecting new bioactive metabolites with biological and pharmacological properties and potential medicinal applications due to their biodiversity, and this is evidenced by the increasing number of publications in the literature [7,8,9,10].

Plants of the genus *Rhizophora* (family Rhizophoraceae) are widely distributed across tropical and subtropical regions where schistosomiasis is endemic, particularly in the mangrove areas of the Fiji, Tonga, and New Caledonia islands, as well as on the coasts of West Africa and Central and South America [11]. In South America, Brazil has the highest incidence and prevalence of schistosomiasis, with approximately 2 million people infected by *S. mansoni* [12]. The secondary metabolites of plants in the genus *Rhizophora* play a role in protection against predators and in adaptive mechanisms to their environment. Within this genus, *R. mangle* is a tree species dominant in Brazilian mangroves, with a latitudinal distribution extending approximately 3700 km along the Brazilian coast [13].

The leaves, stems, roots, and fruits of *R. mangle* have various uses in traditional medicine and exhibit activity against a range of conditions. They have shown efficacy in treating gastric ulcers [14], fatty liver disease and insulin resistance [15], and they have anti-inflammatory and analgesic effects [16]. Additionally, they possess properties for wound healing [17], antioxidant activity [14,18,19], antidiabetic effects [20], and fungicidal and bactericidal actions [19,21,22]. Extracts of *R. mangle* leaves have also demonstrated embryotoxic and embryostatic effects on *Aedes aegypti* eggs and larvae [23], as well as physiological and molluscicidal effects on *Biomphalaria glabrata* (the intermediate host of *S. mansoni*) [24].

The present study aimed to analyze the phytochemical profile of the methanolic extract of *R. mangle* leaves (MELRm) and evaluate its in vitro schistosomicidal activity against adult *S. mansoni* worm couples. This was evaluated through measurements of the mortality, motility, and cell viability of the worms, as well as an evaluation of the extract’s cytotoxicity.

## 2. Results

### 2.1. Chemical Composition of MELRm Extract

Phytochemical analysis of MELRm by UV/Vis spectrophotometry revealed its phenolic composition: flavonoids (215.5 mg QE/g of extract), flavonols (32.0 mg QE/g of extract), tannins (100.3 mg TAE/g of extract), and total phenolic content (423.5 mg GAE/g of extract). In our study, the extract yield was 9.12%. 

**Figure 1:**
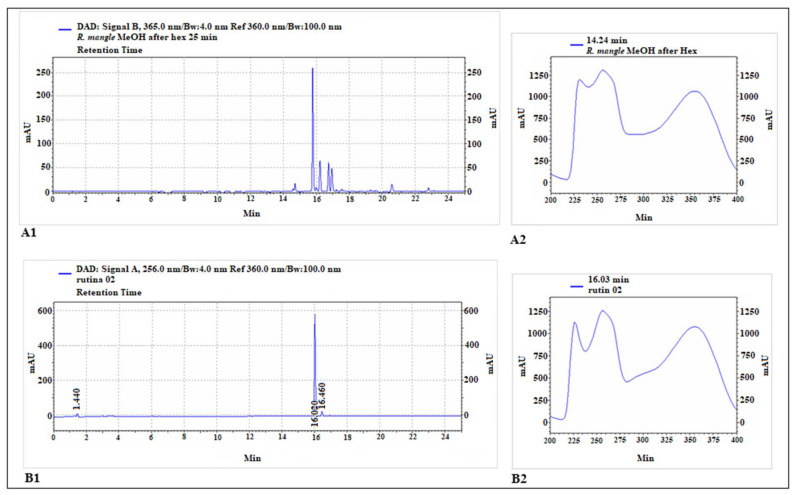
Chromatographic analysis of methanolic extract of *R. mangle* and rutin standard. The main peak of the extract chromatogram (**A1**) exhibited a retention time close to that of rutin (**B1**). The chromophore of this main peak (**A2**) also showed great similarity to that of the rutin standard (**B2**).

The HPLC chromatogram is shown in Figure 1. The constituents identified in MELRm were p-coumaric acid (14.7 min), rutin (15.8 min), ellagic acid (16.2 min), quercetin (16.6 min), apigenin (17.1 min), and geranium (20.6 min), respectively (Table 1). These results highlight that MELRm is predominantly phenolic and indicate the presence of a rutin-like chromophore.

### 2.2. MELRm Induced Mortality, Altered Motility, and Caused Detachment of Adult S. mansoni Worms

Table 2 presents the motility scores of *S. mansoni* at observation intervals of 24, 48, 72, 96, and 120 h following exposure to MELRm. Throughout the observation period (24–120 h), pairs of adult worms incubated in supplemented RPMI 1640 medium (Control 1) or in RPMI 1640 medium with 1% DMSO (Control 2) exhibited normal movement throughout the body, peristalsis of internal organs, and suckers adhered to the bottom or sides of the culture plate, and they maintained the color and integrity of the integument (score 3), indicative of typical in vitro motility. After 24 h of incubation, PZQ (10 µM) resulted in 100% mortality. In this group, no peristaltic or bodily movements were observed (score 0), and mortality occurred while the worms remained paired. The results for the negative and positive control groups are consistent with those reported [25].

MELRm altered motility and caused the separation of worm pairs in the early stages of the experiment. After just 24 h of incubation, the 400 µg/mL dose led to an 81.25% reduction in motor activity, with worms showing movement only in the extremities (anterior and/or posterior regions), the absence of peristalsis, non-adhering suckers (score 1), and the complete separation of worm couples. For this dose, mortality rates of 56.25% and 75% were observed at 72 and 120 h, respectively. At lower doses, in a dose-dependent manner, worms exhibited reduced peristaltic movement along the body, with occasional sucker adhesion (score 2) and the separation of worm couples after 48 h. At 200 and 100 µg/mL, after 72 h of incubation, all worms were uncoupled and showed score 1. Mortality rates at these doses were 62.5% and 50% within 120 h, respectively. At 50 µg/mL, worms did not mate, and changes in motility were observed after 48 h, with half of the worms showing score 1. By the end of the experiment (120 h), 50% of these worms were dead (score 0). At 25 µg/mL, worms maintained score 3 until 72 h of incubation, appearing more agitated than the control groups (Negative Control 1 and 2), with couples beginning to uncouple. By 120 h, at this dose, 100% of the worms had progressed to score 2, and all pairs were fully separated (both males and females).

**Figure 2:**
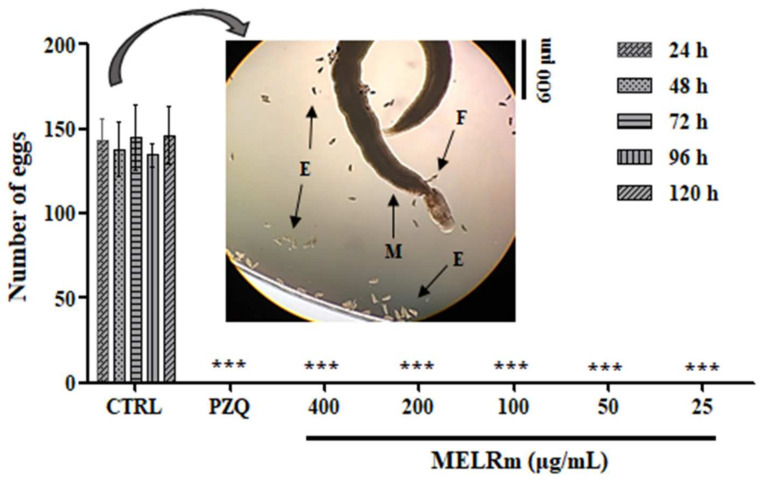
In vitro effect of MELRm on egg production by *S. mansoni* couples. Pairs of parasites (n = 16 worms, male (M), female (F)) were incubated with MELRm at concentrations ranging from 400 to 25 µg/mL for 120 h. Egg (E) counts were assessed using an inverted microscope. Data are expressed as mean ± standard deviation. CTRL = Control 2, RPMI supplemented with 1% dimethyl sulfoxide (DMSO). Statistical significance was denoted as *** *p* < 0.0001 compared to the control.

MELRm effectively inhibited *S. mansoni* oviposition in vitro. Throughout all observation intervals (24, 48, 72, 96, and 120 h) and at all concentrations (400–25 µg/mL), no *S. mansoni* eggs were observed in the culture plates, similar to the results seen with worms exposed to PZQ (Figure 2). In contrast, the worms in the negative control groups (Control 1 and Control 2) produced an average of 140.4 ± 7.33 eggs, with no statistically significant difference between the controls, indicating continued oviposition.

### 2.3. MELRm Induced Cell Death in Adult *S. mansoni* Worms but Showed No Cytotoxicity towards RAW 264.7 Cells

**Figure 3:**
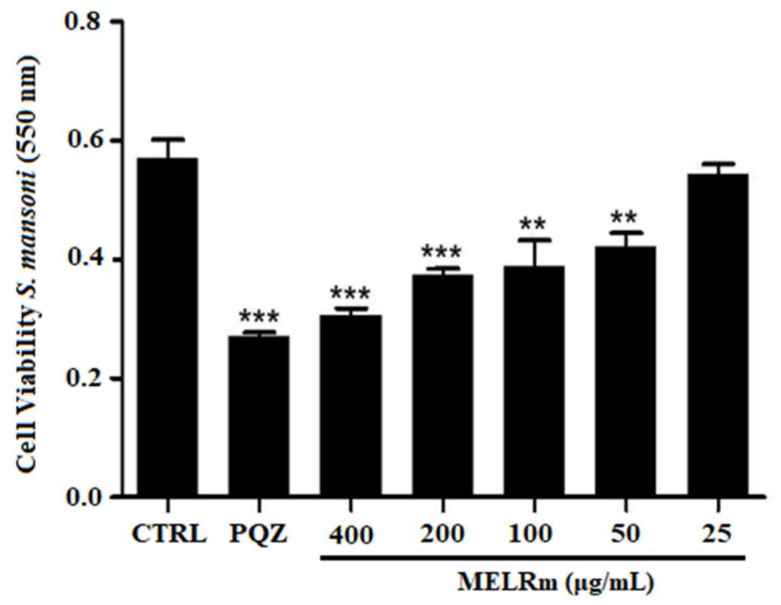
In vitro effects of the methanolic extract of *R. mangle* leaves on the cell viability of *S. mansoni* adult worm couples. Worms in the negative control group (Control 2) were incubated in RPMI 1640 medium with 1.5% DMSO. Positive control worms were treated with praziquantel (PZQ, 10 µM). Cell viability was expressed as the mean ± standard deviation (SD) of absorbance values from four experiments. Statistical significance was denoted as ** *p* < 0.001 and *** *p* < 0.0001 compared to the control.

MELRm significantly reduced mitochondrial viability and, consequently, cell viability in S. mansoni couples, as evidenced by a decrease in formazan crystal formation (Figure 3). MELRm significantly reduced worm cell viability by 31.74% and 26.10% at concentrations of 100 and 50 µg/mL, respectively (*p* < 0.01), compared to the negative control groups. Additionally, concentrations of 200 and 400 µg/mL decreased worm cell viability by 34.55% and 46.45%, respectively (*p* < 0.001). The effects of 200 and 400 µg/mL of MELRm were not significantly different from those of PZQ (*p* > 0.5). DMSO did not affect the cell viability of S. mansoni couples, as formazan crystal formation was similar between Control 1 and Control 2.

**Figure 4:**
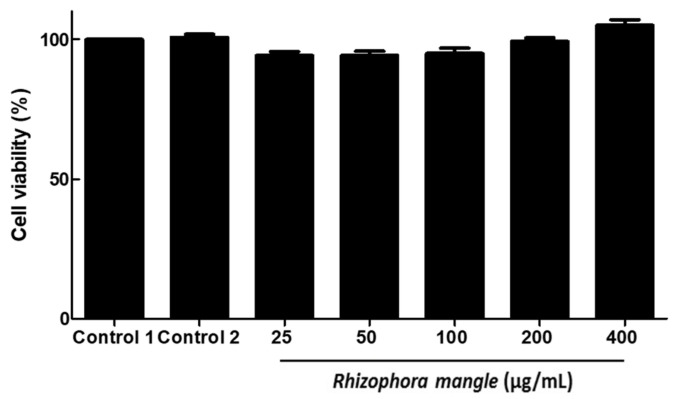
In vitro effects of methanolic extract of *R. mangle* leaves on RAW cell viability.

At the concentrations used in the schistosomicidal evaluation, MELRm did not exhibit cytotoxicity toward RAW cells, as demonstrated in Figure 4.

## 3. Discussion

The emergence of resistance and/or tolerance of *Schistosoma* spp. strains to praziquantel (PZQ), coupled with the rising cost of PZQ and the lack of strategic initiatives for pharmacological interventions against schistosomiasis, has contributed to the inadequacy of current control programs [3]. This underscores the urgent need to develop cost-effective and therapeutic alternatives. In this context, plant-derived products have recently gained prominence as promising sources for new schistosomicidal agents [25,26]. Most studies focusing on screening natural compounds with biological and pharmacological properties have concentrated on terrestrial plants. However, research into marine plants, particularly mangroves, is sparse, despite Brazil’s extensive and underexplored mangrove resources. The authors of [27] emphasize the importance of investigating endemic plant species in regions where schistosomiasis is prevalent. To the best of our knowledge, this study is the first to present UV/Vis spectrophotometry and HPLC data for the methanolic extract of *Rhizophora mangle* (MELRm) collected in Northeast Brazil, along with its in vitro schistosomicidal activity.

Consistent with our findings, secondary metabolites such as terpenoids, tannins, polyphenols, steroids, alkaloids, flavonoids, and saponins have been identified as the primary classes of phytochemicals isolated from mangroves, and they have been characterized for their toxicological and pharmacological properties [8,28,29,30,31]. Our results align with studies reporting phenolic compounds as major constituents in *Rhizophora mangle* leaves collected from Twin Cays, Belize [32], and from Rio de Janeiro, Brazil [33]. An aqueous extract of *R. mangle* bark from Cuban mangroves also contained polyphenols, predominantly tannins, and highlighted the presence of ellagic acid and quercetin. However, unlike our study, additional compounds, such as epicatechin, catechin, chlorogenic acid, and gallic acid, were also identified in this extract [34]. Additionally, it was reported that ethanol, methanol, hexane, butanol, and chloroform extracts of *R. apiculata* leaves exhibited antimicrobial, antioxidant, and anticancer activities, attributed to the presence of phenols and flavonoids [10]. 

Mangroves are biochemically unique ecosystems with high productivity and diversity, offering a wealth of phytochemicals that are of significant interest to the pharmaceutical industry [35]. These plants exhibit a wide range of biological activities, including antibacterial, antioxidant, anticancer, antiseptic, anti-inflammatory, antiulcer, cytotoxic, antiproliferative, insecticidal, larvicidal, antifungal, antidiarrheal, central nervous system depressant, antimitotic, antileukemic, hypoglycemic, and antiplasmodial effects. The broad spectrum of these activities is largely attributed to their rich phytochemical composition, particularly phenolic compounds [28,35,36,37]. Mangrove extracts have been shown to effectively kill larvae of various mosquito species, including Anopheles stephensi (from *Rhizophora apiculata*), *Culex tritaeniorhynchus* (from *Bruguiera cylindrica*), *Aedes aegypti* (from *Rhizophora mucronata*), and *Culex quinquefasciatus* (from *Excoecaria agallocha*) [38]. Additionally, mangroves have commercial applications, serving as sources of wood for export, pigments for tanning leather, charcoal, paper materials, and food products [35,37].

The presence of a rutin-like chromophore in our study does not confirm that rutin is the primary compound in the methanolic extract of *Rhizophora mangle*; it merely indicates a similarity to flavonol-type rutin. Rutin (3′,4′,5,7-tetrahydroxyflavone-3-rutinoside), also known as quercetin-3-O-rutinoside and vitamin P, is a natural polyphenolic flavonoid widely distributed in the plant kingdom [35]. Kandil et al. [32] reported that rutin was the most abundant flavonol glycoside in *R. mangle* leaves from Belize, with significant changes observed during leaf development and senescence. Rutin is known for its extensive pharmacological properties, which have been utilized in human medicine and nutrition [39]. Although the chromatographic profile indicates that rutin is among the primary compounds in MELRm, further analyses are required to fully characterize the chemical profile of this plant and to isolate and investigate its individual constituents.

Extracts and metabolites from medicinal plants used in the treatment of neglected diseases such as leishmaniasis, trypanosomiasis, and schistosomiasis often contain quinones, phenolics, flavonoids, tannins, alkaloids, and terpenes [26,40,41]. Tasdemir et al. [42] investigated the antitrypanosomal and antileishmanial activities of flavonoids and their analogs, revealing that rutin effectively targets the amastigote form of *Leishmania donovani*. They identified rutin as a primary component among the flavonoids in the extract of *Gonocytisus angulatus*. These findings are consistent with [43], who evaluated the HPLC phenolic profile of *Melissa officinalis* and its activity against *L. braziliensis*, *L. infantum*, and *Trypanosoma cruzi*. Their HPLC analysis identified rutin and caffeic acid as principal compounds, and the extract effectively inhibited both promastigote and epimastigote forms, suggesting that these compounds are key to the extract’s antiprotozoal activity. Additionally, quercetin has been shown to exhibit a pro-oxidant effect, generating reactive oxygen species that disrupt the mitochondrial membrane of *L. amazonensis* amastigotes [44,45]. Furthermore, methanolic extracts of *Achillea fragrantissima* rich in flavonoids and tannins demonstrated notable efficacy against *Trypanosoma evansi* [40].

Alemán et al. [46] evaluated the anthelmintic activity of aqueous and methanolic extracts of *R. mangle* against larvae of *Haemonchus* spp. and *Trichostrongylus* spp., noting that 50 mg/mL of either extract induced over 60% mortality in the worms. Moreover, [47] demonstrated the in vitro schistosomicidal activity of the alkaloid saguinarine, which achieved total mortality and significantly reduced cell viability at low concentrations and in brief incubation periods. Recently, an ethanolic extract of *Allium sativum* (garlic) and rutin, a flavonoid derived from garlic, showed promising immunomodulatory and anti-inflammatory effects against *S. mansoni* in a murine model [41]. In this study, the extract reduced the overall worm load, decreased egg accumulation in liver tissue, and increased the elimination of eggs in feces.

In our research, we did not identify any in vitro or in vivo studies reporting the use of *Rhizophora* species against *S. mansoni*. However, the control of schistosomiasis through mangrove plants was documented by Mendes et al. [24]. They explored extracts of *Avicennia schaueriana*, *Laguncularia racemosa*, and *R. mangle* for their molluscicidal activity against *Biomphalaria glabrata*, the intermediate host of *S. mansoni*. Their results highlighted the presence of tannins, alkaloids, triterpenoids, steroids, and coumarins, with *L. racemosa* and *R. mangle* affecting snail motility, feeding, and oviposition, in addition to exhibiting molluscicidal activity. These findings are significant, as the transmission of *S. mansoni* relies on the presence of infected individuals and the contamination of aquatic environments with eggs, which sustain the life cycle of *B. glabrata* and contribute to the chronicity of the infection

Therefore, the development of new schistosomicides that also target oviposition suppression is highly desirable [25,47]. In our study, MELRm induced mortality and motility changes in a dose- and time-dependent manner, leading to the complete separation of worm pairs. Additionally, similar to the PZQ group, no eggs were observed in the culture medium of any groups treated with MELRm throughout the experiment (24–120 h), in contrast to the negative control groups (Control 1 and Control 2). During *S. mansoni* infection, eggs not expelled in feces become embolized and deposited in tissues, primarily in the liver and intestines. These eggs induce a granulomatous inflammatory response, which is a major cause of morbidity and mortality associated with schistosomiasis. While PZQ remains the drug of choice for schistosomiasis, its exact mechanism of action is not fully elucidated, though it is known to cause a rapid influx of calcium, leading to intense spasmodic contractions of the worms’ muscles and resulting in the death of even mated worms [3]. In the case of MELRm, mortality preceded by non-mating may have facilitated the complete exposure of the worms to the extract.

Studies indicate that over 50% of newly approved medications are derived directly or indirectly from plant extracts and/or secondary metabolites, underscoring the importance of identifying new compounds with potential therapeutic activity and low cytotoxicity [48,49,50]. In our study, MELRm did not exhibit cytotoxicity in RAW cells. The RAW 264.7 cell line, a monocyte-/macrophage-like cell line, has been widely used for over 40 years to assess cytotoxicity of various substances. While there is extensive research on the cytotoxicity of extracts and secondary metabolites from mangrove plants, including those of *Rhizophora* species [51,52], we found no prior publications specifically addressing the cytotoxicity of methanolic extracts from *R. mangle* leaves sourced from Northeast Brazil. An aqueous extract of *R. mangle* leaves, bark, and roots has demonstrated an in vitro proliferative effect on HeLa cells [53]. Conversely, the methanolic extract of *R. mangle* leaves, stems, and roots from the Yucatán Peninsula, Mexico, exhibited cytotoxicity in HeLa cells [54]. Compounds isolated from the methanolic extract of *R. apiculata* leaves showed no cytotoxicity against human lung (SK-LU-1), liver (HepG2), and breast (MCF7) cancer cell lines [51]. Additionally, the methanolic extract of *R. mucronata* leaves exhibited selectivity against tumor cell lines without showing cytotoxicity towards healthy cells [55]. It is known that the composition of plant-derived compounds is influenced by soil type, temperature, salinity, and environmental pollution. Furthermore, the choice of solvent for extraction affects the polarity of the metabolites, resulting in variations in biological activity.

Our results do not yet elucidate the potential therapeutic targets and the impact of MELRm on the pathophysiology of schistosomiasis, as this study is limited to in vitro analysis. Nonetheless, our research aligns with the guidelines for initial drug candidate screening, which is a crucial step before advancing to in vivo trials, in accordance with international ethical research standards. Future studies using in vivo experimental models developed by our research group will clarify the effects of MELRm and its fractions on the histopathological and histomorphometric aspects of liver and intestinal tissues, granulomas, cellular and humoral immunoregulation, and parasitological criteria related to *S. mansoni* infection.

## 4. Materials and Methods

### 4.1. Plant Material

**Figure 5:**
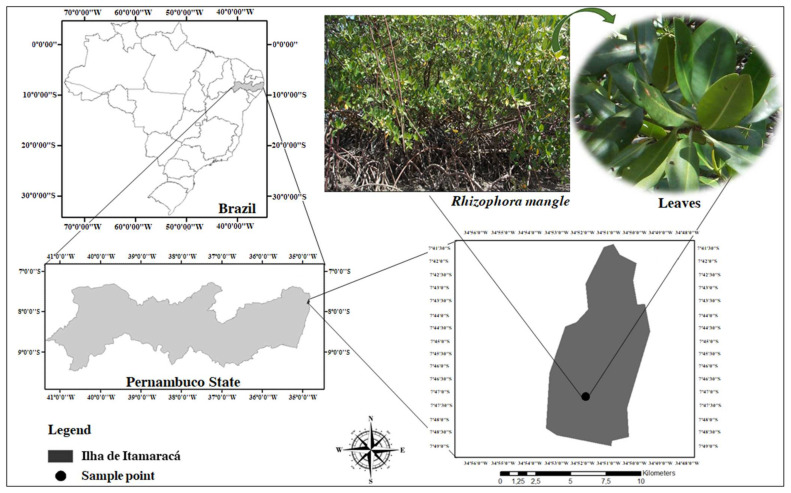
Geographical location of Itamaracá City, Pernambuco, Brazil, indicating the site where *R. mangle* was collected.

*R. mangle* leaves were collected from the mangrove forest in the city of Itamaracá, Vila Velha district, Pernambuco State, Brazil (7°40′ S latitude and 34°50′ W longitude). Only green leaves with a visually intact appearance, free of mechanical damage, pests, diseases, or discoloration, were selected (Figure 5). A voucher specimen, numbered 69,655, was identified by Prof. Dr. Marlene Barbosa, curator of the Herbarium at the Department of Botany, and is deposited in the Herbarium of the Federal University of Pernambuco (UFPE). Collection was authorized by the State Environmental Agency of Pernambuco, Brazil.

### 4.2. Drugs and Reagents

Praziquantel (PZQ) [2 (Cyclohexylcarbonyl) 1,2,3,6,7 11b hexahydro 4H pyrazino [2,1 a]isoquinolin 4 one] (C19H24N2O2, molecular weight 312.41 and purity ≥98%), all analytical-grade solvents, reagents, and materials for cell culture and *S. mansoni* culture were obtained from Sigma Chemical Co., St. Louis, MO, USA.

### 4.3. Extract Preparation

Fresh leaves of *R. mangle* (1 kg) were ground to a particle size of 0.177 mm using a Pulverisette 14 Classic Line knife mill (Fritsch, Pittsboro, NC, USA). The extract was prepared through accelerated solvent extraction (ASE 350, Thermo Scientific, Waltham, MA, USA). Briefly, the crushed material was placed in duplicate into stainless-steel cells in the extractor, each containing 30 g of the material and 100 mL of methanol (Sigma-Aldrich, Darmstadt, Germany) as the mobile phase, under hydrogen pressure (H_2_). The collected *R. mangle* leaf extract (MELRm) was then concentrated under reduced pressure using a rotary evaporator (40.3 °C) (Buchi, New Castle, DE, USA; Vacuum Pump V-700) to remove the methanol. The yield of MELRm was calculated using Equation (1).

(1) $$Yield\;=\;\frac{Extract\;dry\;mass\;(g)}{2aDry\;mass\;of\;leaves\;(g)}\;\times 100\%$$

#### Phytochemical Characterization

The levels of constituents were determined using UV/Vis spectrophotometry based on the methodology proposed by Nerys et al. [56]. The total phenol content was measured using the Folin–Ciocalteau spectrophotometric method. For this assay, 1.0 mL of the extract at a concentration of 500 μg/mL was mixed with 1.0 mL of Folin–Ciocalteau reagent (diluted 1:10 *v*/*v*). After 5 min, 2.0 mL of a 7.5% sodium carbonate (Na_2_CO_3_) solution was added, and after 2 h, the absorbance was measured at 750 nm using a gallic acid standard curve (55 to 550 μg/mL). The results were expressed as milligrams of gallic acid equivalents (GAEs) per gram of extract.

The flavonoid content was determined using a 5 mL solution of 2% aluminum chloride in methanol, which was mixed with an equal volume of the extract solution (500 μg/mL). Absorbance was measured at 415 nm after 10 min. The standard curve was generated using quercetin, and the flavonoid content was expressed as milligrams of quercetin equivalents (QEs) per gram of extract.

The total flavonol content was measured by mixing 2 mL of the extract with 2 mL of 2% AlCl_3_ in ethanol and 3 mL of 50 g/L sodium acetate. The mixture was incubated for 2.5 h at 20 °C. Absorbance was measured at 440 nm, and the result was expressed as milligrams of quercetin equivalents (QEs) per gram of extract using a quercetin standard curve at concentrations ranging from 7.8 to 500 µg/mL. The tannin content was determined by mixing 2 mL of the extract with 3 mL of distilled water and 0.5 mL of Folin–Ciocalteau reagent, allowing it to react for 3 min. This was followed by the addition of 1.5 mL of 17% sodium carbonate (Na_2_CO_3_) and 3 mL of distilled water. The mixture was incubated for 2 h, and absorbance was measured at 725 nm. The equipment blank was prepared using the same proportions of distilled water. Tannin content was expressed as milligrams of tannic acid equivalents (TAEs) per gram of extract, based on a tannic acid standard curve with concentrations ranging from 7.8 to 500 µg/mL.

Characterization was performed using high-performance liquid chromatography (HPLC) with an Agilent 1200 Infinity Series system (Santa Clara, CA, USA), equipped with a quaternary pump, autosampler, column oven, and diode array detector. Separation was achieved on an RP-18 column (Zorbax SB-C18, Agilent, 4.6 × 250 mm, 5 µm). A 5 µL sample was analyzed using a linear gradient at a flow rate of 2.4 mL/min and a temperature of 30 °C, with the following gradient: 98% solvent A (0 min)–90% solvent A (10 min)–15% solvent A (27 min). Solvent A was 0.3% acetic acid in water, and solvent B was acetonitrile. Compound identification was carried out using standards of p-coumaric acid, rutin, ellagic acid, quercetin, apigenin, and geranium under the same experimental conditions used for characterizing the extract. All experiments were conducted in triplicate.

### 4.4. Animals and Ethical Considerations

All experimental procedures were approved by the Ethics Committee on the Use of Animals (CEUA) of the Federal University of Pernambuco (UFPE) under protocol number 0006/2016. Female Swiss Webster mice (*Mus musculus*, 30 ± 2 g) aged 30 days were supplied and housed in the vivarium of the Instituto Keizo Asami (iLIKA/UFPE) under standardized conditions (23 ± 2 °C, 40–50% humidity, and a 12 h light/dark cycle) with free access to water and Labina^®^ food. Strains of *Biomphalaria glabrata* and *S. mansoni* (strain BH, Belo Horizonte, MG, Brazil) are maintained for successive generations in mollusks at the Academic Area of Tropical Medicine at UFPE.

### 4.5. Recovery of Adult Worm Couples

**Figure 6:**
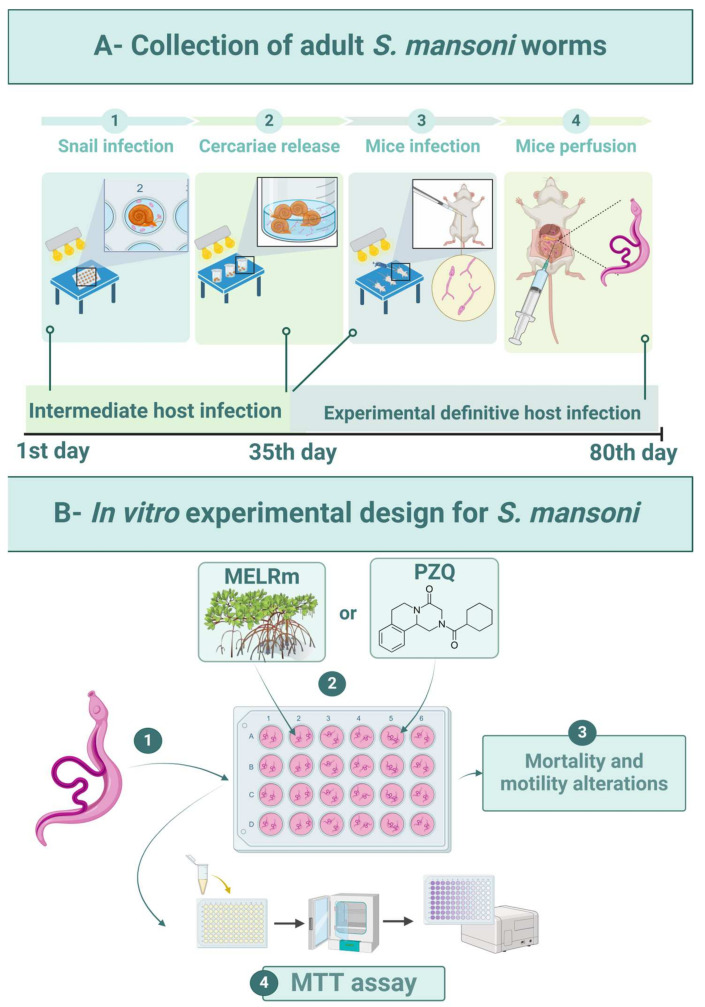
Experimental design. (**A1**)—Experimental infection of *B. glabrata* snails with *S. mansoni* miracidia (BH strain). (**A2**)—Collection of *S. mansoni* cercariae. (**A3**)—Percutaneous infection of mice with cercarial suspension. (**A4**)—Perfusion of mice for worm recovery. (**B1**)—In vitro exposure of worms. (**B2**)—Distribution of MELRm or PZQ. (**B3**)—Evaluation of schistosomicidal activity. (**B4**)—Cell viability assay.

The intermediate hosts were maintained in plastic tanks (50 × 23 × 17 cm) under standardized conditions: filtered and dechlorinated water (20 L) was changed weekly, and the snails were fed daily with *Lactuca sativa* L. and kept at 25 ± 2 °C with a 12 h light/dark cycle. Feces from experimentally infected mice were processed by spontaneous sedimentation to isolate *S. mansoni* eggs. To obtain miracidia, the sedimented feces were exposed to artificial light (60 W, Lightex, Sofia, Bulgaria, model A5570). The infection of adult snails (n = 80) was carried out by placing five miracidia in each well of 24-well culture plates (TPP-Techno Plastic Products, Trasadingen, Switzerland) under artificial light for 4 h (Figure 6(A1)). Afterward, the snails were transferred to tanks and kept shielded from light. Infected snails were exposed to artificial light (60 W) for 2 h to release cercariae after 35 days of infection (Figure 6(A2)).

Mice (n = 10) were infected percutaneously with 120 *S. mansoni* cercariae [57] (Figure 6(A3)). Sixty days after infection, the mice were euthanized by cervical dislocation, and worms were aseptically recovered by perfusion of the hepatic portal system and mesenteric vessels with 0.9% NaCl (*w*/*v*) [58] (Figure 6(A4)). Only intact worm pairs were immediately transferred to RPMI 1640 medium supplemented with 20 mM HEPES, 100 μg/mL penicillin, 100 μg/mL streptomycin, and 10% fetal bovine serum, and they were rinsed four times with this medium.

### 4.6. In Vitro Susceptibility against to S. mansoni

Worms were distributed (two pairs per well) into sterile 24-well culture plates containing 2 mL of supplemented RPMI 1640 medium (Figure 6(B1)). The worms were then incubated at 37 °C in a humidified atmosphere with 5% CO_2_. After two hours of incubation to allow for adaptation, MELRm was added at final concentrations of 25, 50, 100, 200, and 400 μg/mL (Figure 6(B2)). These concentrations were based on previous studies investigating natural compounds against *S. mansoni* [59]. The Negative Control 1 group consisted of worms incubated in supplemented RPMI 1640, while Negative Control 2 contained worms incubated in RPMI 1640 with 1% dimethyl sulfoxide (DMSO). PZQ at a concentration of 10 μM was used as a standard drug and positive control (Figure 6(B2)). PZQ (purity ≥ 98%) was purchased from Sigma Chemical Co. All experiments were performed in quadruplicate (n = 16 pairs of worms per concentration) and repeated at least twice.

#### 4.6.1. Criteria of Schistosomicide Evaluation

##### Mortality and Changes in Motility

An inverted microscope (Leica Microsystems, DM IL, Wetzlar, Germany) was used to evaluate the general condition of the worms, including mortality and changes in motility (Figure 6(B3)). The worms were monitored for five consecutive days at intervals of 24, 48, 72, 96, and 120 h after incubation. The motility and survival of the worms were evaluated according to criteria established by Pica-Mattoccia et al. [60]. Briefly, worms were monitored based on decreasing viability using the following scoring system: score 3—worms exhibited typical movements, including peristalsis of internal organs and movement of suction cups, and were adhered to the bottom or sides of the culture plate (typical for negative control worms); score 2—reduction in overall body movements, peristalsis of internal organs, and suction cups; score 1—movements limited to the extremities or just one extremity (anterior and/or posterior regions), with an absence of peristalsis of internal organs and non-adherent suction cups; and score 0—complete absence of movements, with or without changes in color. The treatment was considered lethal when no parasite movements were observed for up to 2 min. The scoring evaluations were conducted by two double-blind evaluators.

##### Cell Viability Assay of Couples of *S. mansoni* Worms

The viability of *S. mansoni* after treatment was determined using the cytotoxicity assay based on 3-(4,5-dimethylthiazol-2-yl)-2,5-diphenyltetrazolium (MTT) (Figure 6(B4)) [61]. Briefly, two pairs of worms were placed in individual wells of 96-well plates containing 100 μL of MTT (5 mg/mL in phosphate-buffered saline—PBS) and incubated at 37 °C for 30 min. The MTT solution was then replaced with 200 μL of DMSO to dissolve the formazan crystals, and the optical density was measured at 550 nm using a microplate reader (M680, Bio-Rad Laboratories, Inc., Hercules, CA, USA). This procedure was performed with worms from the negative control (1 and 2) and positive control (PZQ) groups under the same experimental conditions. The assay was conducted in quadruplicate and repeated twice.

### 4.7. Cytotoxic Evaluation of MELRm

RAW 264.7 cells were cultured in RPMI 1640 medium supplemented with 2 mM L-glutamine, 1% penicillin-streptomycin, and 10% fetal bovine serum (FBS). The RAW 264.7 cell line (ATCC TIB-71) was obtained from the cell bank of Rio de Janeiro (Brazil) and is maintained in the Cell and Tissue Culture Laboratory of the Department of Histology and Embryology at UFPE. Briefly, cells were seeded into 96-well plates at a density of 1 × 10^6^ cells/well and incubated in a humidified atmosphere of 5% CO_2_ at 37 °C for approximately 48 h, allowing the cells to reach 80–90% confluence. After the incubation period, the cells were washed twice with RPMI 1640 medium. Subsequently, the cells were incubated in RPMI-supplemented medium with different concentrations (25–400 μg/mL) of MELRm for 72 h in a final volume of 200 µL. Next, the culture medium containing MELRm was replaced with 100 µL of MTT solution (5 mg/mL in PBS), and the cells were incubated for 4 h at 37 °C, protected from light. The MTT solution was then removed, and 100 µL of DMSO was added to each well to dissolve the formed purple formazan crystals. Absorbance was measured at 550 nm using a microplate reader (M680, Bio-Rad Laboratories, Inc.). The blank was prepared following the same methodology, but the cells were cultured only in RPMI-supplemented medium [62]. The assays were performed in octuplicate in two independent experiments.

### 4.8. Statistical Analysis

The results were presented as the mean ± SD of the viability percentage from two independent experiments performed in quadruplicate. The median inhibitory concentration (IC50) was calculated from the dose–response curve using GraphPad Prism (San Diego, CA, USA, version 5).

## 5. Conclusions

MELRm is predominantly phenolic and exhibited a chromophore resembling rutin. The extract demonstrated promising schistosomicidal activity, inducing mortality, altering motility, and preventing mating in *S. mansoni* worm couples while also reducing worm cell viability. Importantly, MELRm was found to be non-cytotoxic, confirming its safety. This study underscores the potential of mangrove plants as a valuable natural resource in the search for new therapeutic agents, particularly for the control of *S. mansoni*.

## Figures and Tables

**Table 1:** MELRm extract constituents.

Peak	Compound	Class	Rt, min *
1	p-Coumaric acid	Phenolic acid	14.7
2	Rutin	Flavonoid	15.8
3	Elargic acid	Polyphenol	16.2
4	Quercetin	Flavonoid	16.6
5	Apigenin	Flavonoid	17.1
6	Geranium	Tannin	20.6

* Retention time (minutes).

**Table 2:** Motility scores of control adult worms treated with praziquantel (PZQ) or methanolic extract of *Rhizophora mangle* (MELRm).

Groups Score	Percentage of Worms (%) in Each Motility Score after Incubation																			
	24 h				48 h				72 h				96 h				120 h			
	0	1	2	3	0	1	2	3	0	1	2	3	0	1	2	3	0	1	2	3
Control 1				16 ± 0.0 (100%)				16 ± 0.0 (100%)				16 ± 0.0 (100%)				16 ± 0.0 (100%)				16 ± 0.0 (100%)
Control 2				16 ± 0.0 (100%)				16 ± 0.0 (100%)				16 ± 0.0 (100%)				16 ± 0.0 (100%)				16 ± 0.0 (100%)
PZQ																				
10 µM	16 ± 0.0 (100%)				16 ± 0.0 (100%)				16 ± 0.0 (100%)				16 ± 0.0 (100%)				16 ± 0.0 (100%)			
MELRm μg/mL																				
400		13 ± 1.5 (81.25%)	3 ± 1.0 (18.75%)			16 ± 0.0 (100%)			9 ± 0.5 (56.25%)	7 ± 1.0 (43.75)			11 ± 2.0 (68.75%)	5 ± 1.0 (31.25%)			12 ± 0.5 (75%)	4 ± 0.0 (25%)		
200			6 ± 1.5 (37.5%)	10 ± 2.0 (62.5%)		4 ± 0.0 (25%)	12 ± 0.0 (75%)			16 ± 0.0 (100%)			9 ± 1.5 (56.25%)	7 ± 1.0 (43.75%)			10 ± 0.0 (62.5%)	6 ± 0.0 (37.5%)		
100			4 ± 2.0 (25%)	12 ± 1.5 (75%)			12 ± 1.0 (75%)	4 ± 0.5 (25%)		16 ± 0.0 (100%)			5 ± 1.0 (43.75%)	11 ± 1.5 (56.25%)			8 ± 0.0 (50%)	8 ± 0.0 (50%)		
50				16 ± 0.0 (100%)			8 ± 0.0 (50%)	8 ± 0.0 (50%)		8 ± 0.0 (50%)	8 ± 0.0 (50%)			14 ± 3.0 (87.5%)	2 ± 0.0 (12.5%)			16 ± 0.0 (100%)		
25				16 ± 0.0 (100%)				16 ± 0.0 (100%)				16 ± 0.0 (100%)			5 ± 1.0 (43.75%)	11 ± 1.0 (56.25%)			16 ± 0.0 (100%)	

A total of 16 adult worms (eight pairs) were used per concentration. The experiment was performed in duplicate, totaling 32 worms per concentration. Control 1: Pairs of adult worms incubated solely in supplemented RPMI 1640. Control 2: Pairs of adult worms incubated in RPMI 1640 supplemented with 1.0% DMSO. Motility Scores: Score 3: Typical movements throughout the body, peristalsis of internal organs, and suckers adhered to the bottom and sides of the culture plate. Score 2: Reduced movements throughout the body and decreased peristalsis. Score 1: Movements restricted to the extremities (anterior and/or posterior), with no peristalsis. Score 0: Complete absence of movement and the tegument showing color changes or remaining unchanged.

## Data Availability

The raw data supporting the conclusions of this article will be made available by the authors on request.
